# Incidence and risk factors of tuberculosis among the elderly population in China: a prospective cohort study

**DOI:** 10.1186/s40249-019-0614-9

**Published:** 2020-01-31

**Authors:** Jun Cheng, Yan-Ni Sun, Can-You Zhang, Yan-Ling Yu, Li-Hong Tang, Hong Peng, Ying Peng, Yu-Xia Yao, Shuang-Yi Hou, Jian-Wei Li, Jin-Ming Zhao, Lan Xia, Lin Xu, Yin-Yin Xia, Fei Zhao, Li-Xia Wang, Hui Zhang

**Affiliations:** 1 National Center for Tuberculosis Control and Prevention, Chinese Center for Disease Control and Prevention, Beijing, China; 2 National Center for Population Health and Epidemiology, Canberra, Australia; 3 Heilongjiang Provincial Center for Disease Control and Prevention, Harbin, China; 4 Minhang District Center for Disease Control and Prevention, Shanghai, China; 5 Jiangsu Provincial Center for Disease Control and Prevention, Nanjing, China; 6 Zhejiang Provincial Center for Disease Control and prevention, Hangzhou, China; 7 Henan Provincial Center for Disease control and prevention, Zhengzhou, China; 8 Hubei Provincial Center for Disease Control and Prevention, Wuhan, China; 9 Center for Tuberculosis Control of Guangdong Province, Guangzhou, China; 10 GuangXi Center for Disease Prevention and Control, Nanning, China; 11 Sichuan Provincial Center for Disease Control and Prevention, Chengdu, China; 12 Yunnan Provincial Center for Disease Control and Prevention, Kunming, China

**Keywords:** Tuberculosis, Elderly tuberculosis, Active case finding, Incidence, Risk factor, Follow up, China

## Abstract

**Background:**

China is facing challenges of the shifting presentation of tuberculosis (TB) from younger to elderly due to an ageing population, longer life expectancy and reactivation disease. However, the burden of elderly TB and influence factors are not yet clear. To fill the gap, we generated a cohort study to measure the magnitude of TB incidence and associated factors among the elderly population aged 65 years and above in China.

**Methods:**

In this cohort established in 2013 through a prevalence survey conducted in selected sites, a total of 34 076 elderlies without TB were enrolled into two-year follow-up. We used both active and passive case findings to find out all TB patients among them. The person-year (PY) incidence rates for both bacteriologically positive TB and active TB were calculated. Cox proportional regression model was performed to test effect of risk factors, and the population attributable fraction (PAF) of each risk factor contributing to incident TB among elderlies was calculated.

**Results:**

Over the two-year follow-up period, a total of 215 incident active TB were identified, 62 of which were bacteriologically positive. The incidence rates for active TB and bacteriologically positive TB were 481.8 per 100 000 PY (95% *CI*: 417.4–546.2 per 100 000 PY) and 138.9 per 100 000 PY (95% *CI*: 104.4–173.5 per 100 000 PY), respectively. Incident cases detected by active case finding were significantly higher (*P* < 0.001). Male, non-Han nationality, previously treated TB, ex/current smoker and body mass index (BMI) < 18.5 presented as independent predictors for developing TB disease. For developing bacteriologically positive TB, the biggest contribution was from self-reported ex or current smoker (18.06%). And, for developing active TB, the biggest contribution was from non-Han nationality (35.40%), followed by male (26.80%) and age at 75 years and above (10.85%).

**Conclusions:**

Ageing population in China had a high TB incidence rate and risk to develop TB disease, implying that National TB Program (NTP) needs to prioritize for elderly. Active case finding should be applied capture more active TB cases among this particular population, especially for male, non-Han nationality, and those with identified risk factors.

## Background

Increasing age raises the risk of Tuberculosis (TB). With the increasing longevity and declining fertility rates, the global pace of population ageing is getting faster in the new century [1], and TB in the older adults is becoming clinically important in many countries [2]. It is reported by the World Health Organization (WHO) that TB notification rates in older people (aged 65 years old and above) was higher than other 10-years interval groups in 2016 [3], and many countries witness that the proportion of older TB patients remains high and has even increased in some areas [4], with an increase disproportionately greater than the rise in the number of older persons.

China, a country with the second large TB burden in the world, is challenged by the shifting of TB from younger to elderly due to the ageing population, longer life expectancy and reactivation disease [5–7]. WHO estimated that the percentage of people aged 60 years or over in China is expected to increase from 12.4% (168 million people) in 2010 to 28.0% (402 million) in 2040 [6]. In China, the fifth national TB prevalence survey conducted in 2010 showed the TB prevalence increased with age and peaked in the 75–79 age group, hitting 866/100 000 [5, 8]. According to estimation by Huynh et al. [9], China is unlikely to achieve the incidence target if the National Tuberculosis Program NTP failed to reduce TB incidence among elderlies. Significant impacts of elderly TB control on TB programs had already been observed in Japan, and also in China [10]. If China aims to achieve the milestone of End TB Strategy, the country has to strategically prioritize TB control among the elderlies. There are several approaches recommended to address elderly TB control in China and the choice should be based on TB burden among the elderlies [11]. TB epidemic in this high risk population should be understood and is very helpful for policy making.

Although there are already TB surveillance systems in China, including Infectious Disease Reporting System (IDRS) and TB Information Management System (TBIMS), the real TB incidence rate can not be obtained directly from them. IDRS, an extensive web-based and real-time surveillance system, enables all health facilities across the country to report information on the 37 notifiable diseases (including TB) within 24 h [8]. And, the users of IDRS comprise all TB health facilities, including TB dispensaries and designated hospitals, at province, prefecture and county level [12]. However, TBIMS do not cover non-designated TB facilities, and all information covered by this system are about diagnosed TB patients [13]. A special module in TBIMS acts as an interface between TBIMS and IDRS, and information for one case can be simultaneously updated in these two systems. However, under-reporting of diagnosed TB has also existed in China [14]. A recent retrospective inventory studies conducted in nine counties, by reviewing medical records at the participating health facilities and county-level social insurance system databases, reported that nearly 20% of TB patients were not reported to TBIMS, being higher percentage for children with TB, TB pleurisy, patients diagnosed in the eastern and central regions and patients with a TB diagnosis recorded in either health facilities or social insurance system [14, 15].

Reported TB incidence rate for each age group could be obtained from IDRS, and the reported TB incidence for elderly was two to three times of that for younger adults, being similar difference on TB prevalence rates between these two groups, obtained from the Fifth National Tuberculosis Prevalence Survey [8]. However, this reported incidence underestimated the real status for elderly, because of both the missed cases by surveillance system itself and undiagnosed TB cases resulted from a high percent of asymptomatic TB patients [16]. We established an elderly incidence cohort without TB by conducting a prevalence survey in selected sites in 2013, and utilized both active and passive case findings to capture all incident TB patients during two-year follow-up period, aiming to obtain the real TB incidence rate and identify risk factors for developing TB among elderly.

## Methods

### Cohort establishment from TB prevalence survey in 2013

**Fig. 1:**
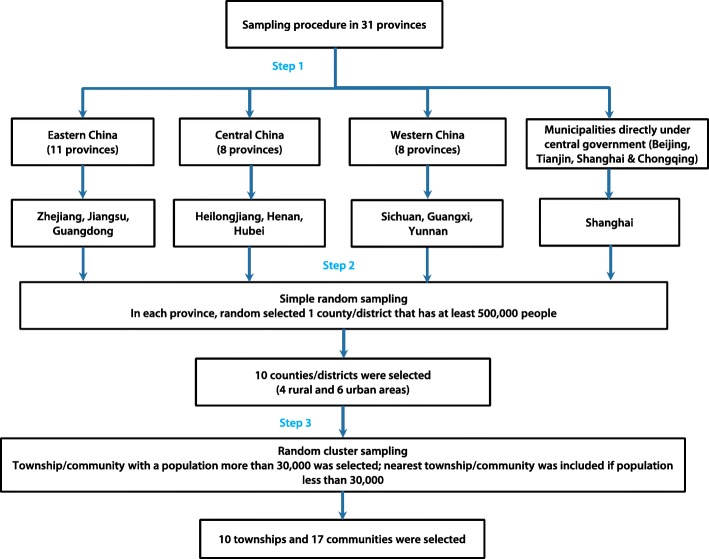
Sampling procedure of the tuberculosis prevalence survey in 2013

We conducted a prevalence survey in selected sites from July to September in 2013, to establish an elderly cohort without TB. The study sites selection and diagnostic algorithm in that prevalence survey has been described fully elsewhere [17]. In brief, the study adopted a three-stage cluster and random sampling for selecting study sites (Fig. 1), and finally 10 townships and 17 communities located in 10 counties covering 38 888 elderly were selected. For each elderly, door-to-door interview for data collection, physical examination for calculating body mass index (BMI), and chest X-ray (CXR) examination were provided for them. Those elderlies who had suspected TB symptom or abnormal chest radiography were required to provide three sputum specimens (one “at spot”, one in early morning and one at night) for laboratory diagnosis. Finally, 4619 refused to participate this prevalence survey, leaving 34 269 elderlies finishing all required procedure, and 193 active TB cases were identified.

The information obtained from door-to-door interview, including demographic, most of disease history, lifestyle and suspected TB symptoms, were totally self-reported by participants, except for history of diabetes and treated TB previously. For diabetes information, we also checked their personal health document covered by the National Basic Public Health Service Project and updated by year. Participants self-reporting diabetes history or with a medical record for diabetes diagnosis were classified as known diabetes patients. For retreated TB patients, we checked TBIMS to identify whether participants were diagnosed with TB and finished treatment or cured, and either self-reporting or recorded as a treated TB patient previously was classified as retreated TB cases.

### Sample size and study population

The sample size was calculated for a population cohort study. TB incidence rates for general population with TB history and without TB history were 2706.3 per 100 000 person-year (PY) and 39.6 per 100 000 PY, respectively, obtained from another cohort study in China [18]. Assuming TB incidence rate among elderly being two times of that for general population, we used 5412.6/100 000 person-year and 79.2/100 000 person-year as the estimated TB incidence for elderly with TB history (P_1_) and that for elderly without TB history (P_2_). The study was set at a power of 0.8 (one-side) and two-tailed significance of 0.05. Based on the formula, a total of 146 elderly with TB history should be recruited. Assuming the percentage of persons with TB history in elderly was 20% higher than that in general population (0.42% obtained from cohort study in China [18]), the number of elderly needed to registered into cohort should be 28 765. Considering a 15% non-response rate and lost to follow up, we increased the sample size to 33 080. In the 27 study sites, people who were born before September 31, 1948 (aged 65 years and older at the time of implementing baseline prevalence survey), continuously lived in the village or community for six-month and longer were eligible for our prospective study. Excluding 193 active TB identified in baseline prevalence survey, 34 076 elderlies without TB were enrolled into our incidence study.

$$ N=\frac{{\left[{Z}_{\alpha}\sqrt{2\overline{p}\left(1-\overline{p}\right)}+{Z}_{\beta}\sqrt{p_1\left(1-{p}_1\right)+{p}_2\left(1-{p}_2\right)}\right]}^2}{{\left({p}_1-{p}_2\right)}^2} $$

### Field procedure

Our study population enrolled at baseline was set as a fixed cohort during this prospective study period, that is, only elderly enrolled into our incidence study at baseline will be followed up, and, those uncovered at baseline and met inclusion criteria during the two-year follow-up period were excluded from our analysis.

The informed consent was obtained from all participants before the door-to-door interview at baseline survey. For elderlies with difficulties in communication, his/her family members were interviewed as the respondent. During the two-year follow-up period, for those who were diagnosed as active TB cases, the information of TB occurrence and date were immediately recorded, and further identified in TBIMS. And, every year for the follow-up period, we conducted a door-to-door investigation to identify the follow-up status for each participant, and promote them to participate required chest radiography examination. Meanwhile, the information of other outcomes, including death, transfer out or move, and the date of outcome occurrence were asked and recorded accurately.

From October 2013 to September 2015, we conducted both active and passive case finding annually to detect and diagnose incident TB patients among this study cohort. For active case finding, the same procedure and diagnostic algorithm as baseline prevalence survey were used, that is, TB symptom screening and chest radiography examination were provided for each sampled elderly, and sputum smear microscopy and culture were performed for those with TB symptoms or abnormal chest radiography. For passive case finding, we asked detailed TB symptoms information among elderlies who actively visited township hospitals and village clinics during the study period. CXR and collection of sputum specimen were administered to those with TB suspected symptoms. If there were any TB cases reported during the study period, we collected their diagnosis and date. For those being diagnosed as TB cases by active case finding or passive case finding, treatment was arranged by local hospitals.

### Study outcome and definition

We measured the study outcomes as incident active TB, death, transfer and moving out from study sites, and refusal to receive yearly chest radiography during the follow-up period. We observed and recorded whichever came first as an endpoint in the study according to the door-to-door interview, passive case finding and yearly screening.

Active TB included bacteriologically positive TB and clinical diagnosed TB. A bacteriologically positive TB patient was defined as an individual with at least one sputum smear or culture positive. Smear positive was that the sputum specimen had at least one acid-fast bacillus identified within 100 fields under microscopy. Culture positive was that the specimen had at least one colony of *Mycobacterium tuberculosis* complex being isolated by using Löwenstein-Jensen medium [19]. The clinical diagnosed TB was defined by the presence of three negative smears and CXR abnormalities consistent with active pulmonary TB, and meeting at least one of the following criteria: clinical TB symptoms, or strong positive purified protein derivative reaction, or TB lesions confirmed by histopathological examination of extrapulmonary tissue, or excluding other pulmonary disease by diagnostic treatment or follow-up observations [19]. The elderly who moved out and did not come back during the study period was recorded as transferred or moved out. Refusal was defined as an elderly who refused to provide information, or to finish all requested TB check procedure. Moved or transferred out and refusal were categorized as lost-to-follow-up in analysis of this study.

### Quality control

Strict quality control was performed across the whole study process. The questionnaire interview was carried out among all eligible elderly face to face by trained staff at county level. Questionnaires collected were checked by data management specialist for completeness and logicality. After completing data entry, 2% of questionnaires were randomly selected for consistency check by the national expert group. If discrepancies identified, information was revised on the second day by asking the responsible interviewer. If the interviewer could not work out the discrepancy, enquires were made to local staff to check with the respondent. All chest radiography films and sputum specimens collected were transported to TB designated hospital at county level for diagnosis confirmation and strain identification. All chest radiography films for TB cases were reviewed by a national expert group within two months after yearly follow-up completion. The smear positive TB was assessed and classified strictly by trained laboratory technicians at county level according to the national standardized methods and procedure. The quality of culture conducted in county laboratory was supervised and monitored by prefectural or provincial TB laboratory. All the sputum smear and culture results were checked by the staff from the National Tuberculosis Reference Laboratory for quality control. In addition to the rigorous internal quality control for data collection, this study also had an independent site auditing team from WESTAT (Rockville, MD, USA). The field inspectors from WESTAT went to study sites regularly to check if the procedure performed followed the research legislation in terms of consent, progress, storage and maintenance of records by working with the research team in the field to ensure study procedure being in line with the study design and guarantee the data quality in terms of completion and authenticity.

### Data management and statistical analysis

We developed a web-based dataset for data entry and management. All data collected from baseline and follow-up surveys were double entered. Data clean and consistency check were performed by the national study team. Statistical inference followed standard procedure for data analysis. Pearson’s *χ*^2^ test or Fisher’s exact test was used to test for differences in proportions. Two-sided *P*-values of < 0.05 were considered significant. The TB incidence rates for bacteriologically positive and active TB were calculated and expressed as cases per 100 000 PY. The follow-up period, from the date of enrolment to the occurrence of incident TB, death, transfer or moving out, completion of follow-up or the end of the study, was computed as the time passed. PY for each participant in this cohort was calculated based on individual information, that is, the interval of finishing baseline survey date and the exact date of outcome occurrence was calculated one by one and considered as his/her PY.

Univariate cox proportional regression model was performed with time dependent covariate in relation to TB incidence using a forward inclusion approach. Demographic character, yearly household income per head, history of diseases, smoking and drinking status, and BMI at baseline were regarded as influence factor and included into our analysis. We classified those with less than RMB 2300 income per year as low-income group according to the national poor standard (2011), and for those with more income, we divided them into two groups by cutoff point being RMB 10 000. According to disease history, participants was classified into yes or no or unknown groups. We categorized BMI as underweight (BMI < 18.5 kg/m^2^), normal weight (≥ 18.5 kg/m^2^ and < 24.0 kg/m^2^), overweight and obese (≥ 24.0 kg/m^2^) [20]. Variables significantly correlated in the univariate model and of epidemiological interest were included in the multivariate model. Hazard ratios (HR) and 95% confidence intervals (*CI*) were used to assess the risk of developing TB. Data analysis was performed using SPSS software (version 17.0, SPSS for Windows Release 17.0, SPSS Inc., Chicago, Illinois, USA).

Based on the results from multivariate analysis, we calculated the population attributable fraction (PAF) of each risk factor contributing to incident TB among elderlies. We used the adjusted HR and baseline prevalence of each associated factor among elderlies to estimate its contribution to bacteriologically positive and active TB, respectively.

### Ethical Approval

The study protocol was approved by the Research Ethics Review Committee of Chinese Center for Disease Control and Prevention (Approval number: 201322). The signed informed consent was obtained from all participants before investigation.

## Results

### Elderlies followed up between 2013 and 2015

**Fig. 2:**
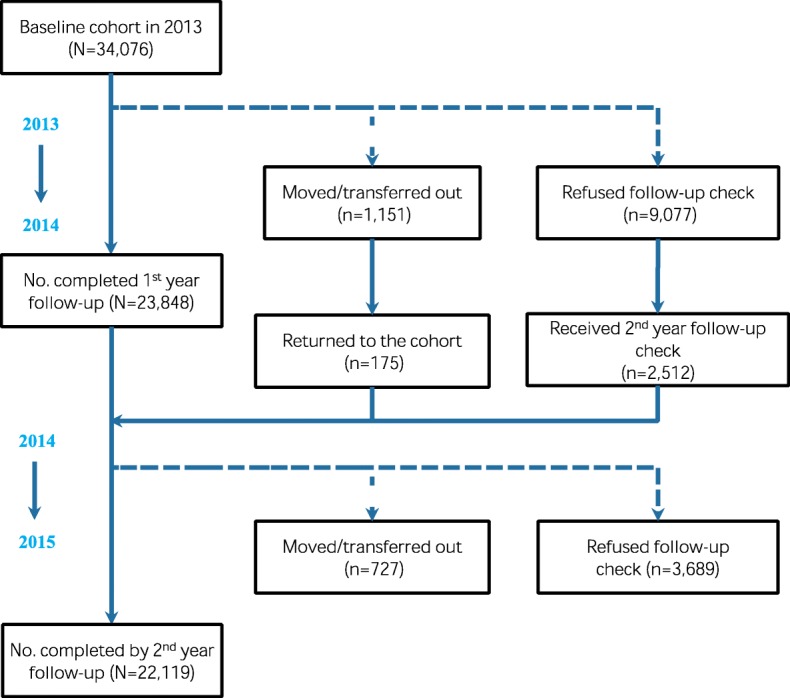
Number of elderly people followed up, 2013–2015

Among the 34 269 elderly people finishing baseline prevalence survey, 193 were detected as active TB cases, leaving 34 076 elderlies in the two-year follow-up. At the end of follow-up period, 22 119 elderly people remained in the cohort for data analysis (Including those transferred out or moved, or refused follow-up check in the first follow-up year, and finished the second year follow-up check) (Fig. 2).

### Demographic characteristics of the follow-up cohort compared to the lost-follow-up cohort

The mean age of the study cohort was 73.0 (± 6.36 years old, ranged from 65 to 108 years old) and about two third were aged from 65 to 74 years. We compared the demographic characteristics of the elderly cohort followed up with those lost to follow-up during the study period, given the high proportion of elderlies deceased, refused to follow-up examination and moved or transferred out (Table 1). There were significant more elderlies aged 75 and above (40.3%, *P* < 0.001) lost to follow up. Of the lost to follow-up cohort, most elderly (86.7%) were Han nationality (*P* < 0.001). A higher proportion of lost to follow up presented in elderlies with higher education (6.1% vs 19.4%; *P* < 0.001). Elderlies without spouse had higher proportion of lost to follow-up (*P* < 0.01). A greater proportion of elderlies who were not local residents lost to follow up during the study period (17.8% versus 2.9%; *P* < 0.001). A substantial proportion (64.9%) of elderly people who reported higher annual average income lost to follow up (*P* < 0.001). About 10% of the cohort had a BMI less than 18.5, and a greater proportion with BMI ≥ 24 lost to follow up (*P* < 0.001).

**Table 1:** Comparison of characteristics between the follow-up and lost-to-follow up cohorts, 2013–2015*

Variables	Total	Follow-up	Lost-to-follow up	*P*
	*N* (%)	*n* (%)	*n* (%)	
Total	34 076	22 119	11 957	
Gender				
Male	15 913 (46.7)	10 385 (47.0)	5528 (46.2)	0.207
Female	18 163 (53.3)	11 734 (53.0)	6429 (53.8)	
Age groups				
65–74	21 592 (63.4)	14 452 (65.3)	7140 (59.7)	< 0.001
≥ 75	12 484 (36.6)	7667 (34.7)	4817 (40.3)	
Nationality				
Han	29 554 (86.7)	18 918 (85.5)	10 636 (89.0)	< 0.001
Others	4507 (13.3)	3198 (14.5)	1309 (10.9)	
Unknown	15(0.1)	3 (0.0)	12 (0.1)	
Education				
Illiteracy	12 016 (35.3)	8645 (39.1)	3371 (28.2)	< 0.001
Primary to secondary	17 168 (50.4)	11 413 (51.6)	5755 (48.1)	
High school and above	3663 (10.7)	1343 (6.1)	2320 (19.4)	
Unknown	1229 (3.6)	718 (3.2)	511 (4.3)	
Marital status				
Married	24 828 (72.9)	15 970 (72.2)	8858 (74.1)	0.001
Others#	9244 (27.1)	6147 (27.8)	3097 (25.9)	
Unknown	4 (0.0)	2 (0.0)	2 (0.0)	
Residence				
Local resident	31 316 (91.9)	21 487 (97.1)	9829 (82.2)	< 0.001
Others	2760 (8.1)	632 (2.9)	2128 (17.8)	
Annual average income per person in the family (RMB)				
≥ 10 000	16 506 (48.4)	8748 (39.5)	7758 (64.9)	< 0.001
2300–9999	11 935 (35.0)	9230 (41.7)	2705 (22.6)	
< 2300	4010 (11.8)	3058 (13.8)	952 (8.0)	
Unknown	1625 (4.8)	1083 (5.0)	543 (4.5)	
Diabetes				
No	31 690 (93.0)	20 697 (93.6)	10 993 (91.9)	< 0.001
Yes	2386 (7.0)	1422 (6.4)	964 (8.1)	
Previously treated with TB				
No	33 503 (98.3)	20 530 (98.4)	11 750 (98.3)	0.367
Yes	573 (1.7)	333 (1.6)	207 (1.7)	
Chronic bronchitis				
No	32 346 (94.9)	20 905 (94.5)	11 441 (95.7)	< 0.001
Yes	1632 (4.8)	1147 (5.2)	485 (4.1)	
Unknown	98 (0.3)	67 (0.3)	31 (0.3)	
Pneumoconiosis				
No	33 989 (99.7)	22 052 (99.7)	11 937 (99.8)	0.002
Yes	77 (0.2)	63 (0.3)	14 (0.1)	
Unknown	10 (0.0)	4 (0.0)	6 (0.1)	
Cigarette smoker				
Non-smoker	27 363 (80.3)	17 544 (79.3)	9819 (82.1)	< 0.001
Ex/current smoker	6707 (19.7)	4571 (20.7)	2136 (17.9)	
Unknown	6 (0.0)	4(0.0)	2(0.0)	
Alcohol drinker				
Non-drinker	27 526 (80.9)	17 604 (79.6)	9922 (83.0)	< 0.001
Ex/current drinker	6501 (19.1)	4499 (20.3)	2002 (16.7)	
Unknown	49 (0.1)	16 (0.1)	33 (0.3)	
BMI level				
18.5–23.9	20 754 (60.9)	13 680 (61.8)	7074 (59.2)	< 0.001
< 18.5	3585 (10.5)	2477 (11.2)	1108 (9.3)	
≥ 24	9730 (28.6)	5958 (26.9)	3772 (31.5)	
Unknown	7 (0.0)	4 (0.0)	3 (0.0)	

# Others included single, divorced and widowed. *TB* Tuberculosis, *BMI* Body mass index

### Incidence rate of bacteriologically positive TB and associated risk factors among elderly

Over the follow-up period, the observed cumulative PY for the elderly cohort was 44 622.2. A total of 62 bacteriologically positive incident TB cases were identified and the incidence rate was 138.9 per 100 000 PY (95% *CI*: 104.4–173.5 per 100 000 PY). Among the 62 incident cases, 79.0% (49) were detected by active case finding, compared to 21.0% (13) by passive case finding (*P* <  0.001). The incidence rate for elderly with different characteristics and HRs were calculated and presented in Table 2. Those elderlies, who were male, aged older than 75 years, Han nationality, illiteracy, married, living outside the study areas, annual average income per person in the family less than RMB 2300, previously treated with TB, suffering chronic bronchitis, ex−/current smoker and BMI less than 18.5 had a much higher TB incidence rates over the follow-up period.

**Table 2:** Incidence rate of bacteriologically positive TB and associated risk factors among the elderly followed up, 2013–2015^#^

Variables	Person-year	TB cases (*n*)	Incidence(1/100 000 person-year)(95% *CI*)	HRc (95% *CI*)	HRa (95% *CI*)	*P*
Total	44 622.2	62	138.9 (104.4–173.5)			
Gender						
Female	23 959.1	23	96.0 (56.8–135.2)	1.00	1.00	
Male	20 663.1	39	188.7 (129.5–248.0)	1.97 (1.18–3.30)**	1.47 (0.80–2.69)	0.210
Age group						
65–74	28 910.3	33	114.2 (75.2–153.1)	1.00	1.00	
≥ 75	15 711.9	29	184.6 (117.4–251.8)	1.63 (0.99–2.69)	1.50 (0.90–2.48)	0.117
Nationality						
Han	38 909.5	61	156.8 (117.4–196.1)	1.00		
Others	5703.3	1	17.5 (0.4–97.7)	0.21 (0.03–1.52)		
Education						
High school and above	3536.9	4	113.1 (30.82–289.5)	1.00		
Primary to secondary	23 111.4	30	129.8 (83.4–176.3)	0.81 (0.29–2.32)		
Illiteracy	16 550.9	28	169.2 (106.5–231.8)	1.19 (0.42–3.40)		
Marital status						
Married	32 515.4	49	150.7 (108.5–192.9)	1.00		
Others^$^	12 101.9	13	107.4 (49.0–165.8)	0.77 (0.42–1.42)		
Residence						
Local residents	42 495.9	58	136.5 (101.4–171.6)	1.00		
Others	2126.3	4	188.1 (51.3–481.6)	2.46 (0.89–6.84)		
Annual average income per person in the family (RMB)						
≥ 10 000	19 617.4	22	112.2 (65.3–159.0)	1.00		
2300–9999	17 234.1	28	162.5 (102.3–222.6)	1.35 (0.77–2.36)		
< 2300	5708.9	12	210.2 (91.3–329.1)	1.95 (0.96–3.96)*		
Diabetes						
No	41 653.3	62	148.9 (111.8–185.9)	1.00		
Yes	2968.9	0	0.0 (0–124.29)	0.04 (0.00–3.73)		
Previously treated with TB						
No	43 871.8	60	136.8 (102.2–171.4)	1.00		
Yes	750.4	2	266.5 (32.3–962.2)	1.70 (0.42–6.97)		
Chronic bronchitis						
No	42 311.8	58	137.1 (101.8–172.4)	1.00		
Yes	2188.2	4	182.8 (49.8–468.0)	1.26 (0.46–3.49)		
Pneumoconiosis						
No	44 500.5	62	139.3 (104.6–174.0)	1.00		
Yes	112.6	0	0.0 (0.0–3275.9)	-#		
cigarette smoker						
Non-smoker	35 608.9	38	106.7 (72.8–140.7)	1.00	1.00	
Ex/current smoker	9006.3	24	266.5 (159.9–373.1)	2.27 (1.36–3.78)**	2.12 (1.27–3.54)	0.004
alcohol drinker						
Non-drinker	35 752.6	48	134.3 (96.3–172.2)	1.00		
Ex/current drinker	8827.0	14	158.6 (75.5–241.7)	1.06(0.58–1.92)		
BMI level						
18.5–23.9	27 380.2	39	142.4 (97.7–187.1)	1.00	1.00	
< 18.5	4789.2	17	355.0 (186.2–523.7)	2.37 (1.34–4.19)**	2.33 (1.32–4.12)	0.004
≥ 24	12 445.2	6	48.2 (17.7–104.9)	0.33 (0.14–0.77)**	0.34 (0.14–0.80)	0.014

Those with missing values were excluded from analysis; *TB* Tuberculosis, *HRc* crude hazard ratio; *HRa* hazard ratio adjusted; *P ≤ 0.05; ** *P* < 0.01; *** *P* < 0.001. ^$^Others included single, divorced and widowed. # Unavailable HRc because of no case for this group

In the univariate model, elderlies who were male, ex or current smoker and BMI < 18.5 had higher risk in developing incident TB. BMI ≥ 24 presented as a protection factor for developing bacteriologically positive TB disease. All statistically significant variables in univariate analysis were included into multivariate analyses to calculate the adjusted HR. In the multivariate model, ex or current smoker (HR = 2.12, 95% *CI* 1.27–3.54; *P* < 0.01) and BMI < 18.5 (HR = 2.33, 95% *CI*: 1.32–4.12; *P* < 0.01) were the strong predictors in developing bacteriologically positive TB disease. For those elderlies with BMI ≥ 24.0, their risks in developing bacteriologically positive TB were substantially reduced by 66%.

### Incidence rate of active TB and associated risk factors among elderly

During the study period, a total of 215 active TB cases were detected and the incidence rate was 481.8 per 100 000 PY (95% *CI*: 417.4–546.2 per 100 000 PY) (Table 3). Among the 215 active cases, 83.7% (180) were detected by active case finding, compared to 16.3% (35) by passive case finding (*P* < 0.001). Those elderlies, who were male, aged 75 years and older, non-Han nationality, illiteracy, married, local residents, annual average household income per person less than RMB 2300, not diabetes, previously treated with TB, chronic bronchitis, pneumoconiosis, ex or current smoker, ex-drinker or current drinker and BMI < 18.5 had higher incidence rates.

**Table 3:** Incidence rates and relative risk for active TB among elderlies followed up, 2013–2015^#^

Variables	Person-year	TB cases (*N*)	Incidence(1/100 000 person-year (95% *CI*)	HRc (95% *CI*)	HRa (95% *CI*)	*P*
Total	44 622.2	215	481.8 (417.4–546.2)			
Gender						
Female	23 959.1	76	317.2 (245.9–388.5)	1.00	1.00	
Male	20 663.1	139	672.7 (560.9–784.5)	2.11 (1.59–2.80)***	1.78 (1.27–2.50)	0.001
Age group						
65–74	28 910.3	121	418.5 (344.0–493.1)	1.00	1.00	
≥ 75	15 711.9	94	598.3 (477.3–719.2)	1.42 (1.08–1.87)*	1.33 (1.00–1.80)	0.053
Nationality						
Han	38 909.5	157	403.5 (340.4–466.6)	1.00	1.00	
Others	5703.3	58	1017.0 (755.2–1278.7)	5.29 (3.77–7.41)***	5.15 (3.52–7.54)	< 0.001
Education						
High school and above	3536.9	12	339.3 (147.3–531.3)	1.00		
Primary to secondary	23 111.4	108	467.3 (379.2–555.4)	1.00 (0.55–1.82)		
Illiteracy	16 550.9	95	574.0 (458.6–689.4)	1.36 (0.74–2.49)		
Marital status						
Married	32 515.4	168	516.7 (438.6–594.8)	1.00		
Others^$^	121 01.9	47	388.4 (277.3–499.4)	0.82 (0.59–1.13)		
Residence						
Local residents	42 495.9	207	487.1 (420.8–553.5)	1.00		
Others	2126.3	8	376.2 (162.3–741.2)	1.44 (0.71–2.92)		
Annual average income per person in the family (RMB)						
≥10 000	19 617.4	63	321.1 (241.8–400.5)	1.00	1.00	
2300–9999	17 234.1	93	539.6 (430.0–649.3)	1.53 (1.11–2.11)***	1.32 (0.95–1.83)	0.095
< 2300	5708.9	41	718.2 (498.4–938.0)	2.29 (1.54–3.40)***	1.48 (0.97–2.26)	0.072
Diabetes						
No	41 653.3	206	494.6 (427.0–562.1)	1.00		
Yes	2968.9	9	303.1 (138.8–575.3)	0.62 (0.32–1.21)		
Previously treated with TB						
No	43 871.8	201	458.2 (394.8–521.5)	1.00	1.00	
Yes	750.4	14	1865.7 (888.4–2843.0)	3.70 (2.15–6.37)***	3.18 (1.83–5.52)	< 0.001
Chronic bronchitis						
No	42 311.8	199	470.3 (405.0–535.7)	1.00		
Yes	2188.2	16	731.2 (372.9–1089.5)	1.55(0.93–2.59)		
Pneumoconiosis						
No	44 500.5	213	478.7 (414.4–542.9)	1.00		
Yes	112.6	2	1755.6 (214.8–6409.8)	3.65 (0.91–14.71)		
cigarette smoker						
Non-smoker	35 608.9	146	410.0 (343.5–476.5)	1.00	1.00	
Ex/current smoker	9006.3	69	766.1 (585.4–946.9)	1.77 (1.33–2.37)***	1.48 (1.06–2.09)	0.023
alcohol drinker						
Non-drinker	35 752.6	156	436.3 (367.9–504.8)	1.00	1.00	
Ex/current drinker	8827.0	59	668.4 (497.9–839.0)	1.37 (1.01–1.85)*	0.941 (0.66–1.34)	0.732
BMI level						
18.5–23.9	27 380.2	147	536.9 (450.1–623.7)	1.00	1.00	
< 18.5	4789.2	37	772.6 (523.6–1021.5)	1.39 (0.97–2.00)	1.29 (0.88–1.88)	0.194
≥ 24	12 445.2	31	249.1 (161.4–336.8)	0.46 (0.31–0.67)***	0.53 (0.35–0.79)	0.002

#Those with missing values were excluded from analysis; *TB* Tuberculosis, *HRc* crude hazard ratio, *HRa* hazard ratio adjusted; *P ≤ 0.05; ** *P* < 0.01; *** *P* < 0.001; ^$^Others included single, divorced and widowed

In univariate analyses, male, age group at 75 years and older, non-Han nationality, annual average household income per person less than RMB 10 000 Yuan, previously treated with TB, self-reported smoker and drinker presented statistically significant risk of developing active TB among elderly. BMI ≥ 24 was a statistically significant protection factor for elderlies from developing active TB. In the multivariate model, male, non-Han nationality, previously treated TB and ex or current smoker were independently associated with the increased risk of developing active TB. And, BMI ≥ 24 decreased the risk of developing active TB by 47%.

### Population attributable factors to TB incidence among elderlies followed up

We calculated the PAF of identified risk factors for TB using adjusted HR obtained from multivariate analyses (Table 4). For bacteriologically positive TB, self-reported ex or current smoker had the biggest contribution (18.1%) followed by male (18.0%) and age at 75 years and above (15.4%). For active TB, non-Han nationality (35.4%) had the biggest contribution followed by male (26.8%) and age at 75 years and above (10.8%).

**Table 4:** Contribution of risk factors for bacteriologically positive TB and active TB

Risk factors	Prevalence among cohort	Bacteriologically positive TB		Active TB	
		HRa	PAF (%)	HRa	PAF (%)
Male	0.467	1.470	18.0	1.784	26.8
Age(≥ 75)	0.366	1.498	15.4	1.332	10.8
Other nationality	0.132	-	-	5.152	35.4
Previously treated with TB	0.017	-	-	3.175	3.6
Ex/current smoking	0.197	2.119	18.1	1.484	8.7
BMI < 18.5	0.105	2.328	12.2	1.285	2.9

-: Not applicable; *TB* Tuberculosis, *PAF* Population attributable fraction, *HR* Hazard ratio, *BMI* Body mass index;

## Discussion

The high TB incidence obtained from our prospective cohort study highlighted the importance of active case finding among elderly in China. The results indicated that male, non-Han nationality, previously treated with TB, ex/current smoker and BMI < 18.5 were risk factors for developing TB disease among elderly in China.

TB incidence is a key indicator to evaluate TB epidemic and the effectiveness of control strategy at country level as set in the END TB strategy by WHO [21–23]. Although cohort study, as a kind of prospective study, had advantages of identifying causality and yielding incidence, this direct measurement of TB incidence is prohibitively complicated both logistically and financially, therefore, indirect estimation of TB incidence is widely used [23]. For countries with reliable surveillance system, directly notification rate is used as incidence rate, including reporting the epidemiological status for elderly TB. In the literature we reviewed, TB incidence rate among elderlies was various in countries with high and low TB burden. For the United States and Germany, the reported average yearly rate of TB disease among aged 65 years and above was 10.9 per 100 000 [24] and 11.2 per 100 000 [25], respectively. In South Africa, the reported incidence rate was 518–684 per 100 000 for male and 193–314 per 100 000 for female among people aged 65 years and older [26]. An eight-year follow-up study conducted among the elderlies in Taiwan Province reported similar incidence rate (175.5 per 100 000 person-year) [27] to our study. The TB incidence rate we reported was higher than the rate reported by using IDRS data (about 155/100000 for the elderlies) due to the implementing of yearly active casing finding.

Elderlies are generally at risk to develop TB due to compromised immune responses and reactivation of previous “latent TB” or new TB infection [25, 28, 29]. Our study identified that age, male [30], smoking [31], previously treated TB, BMI < 18.5 [32] and low annual household income as main risk factors for elderly TB, being very similar to other studies globally [30–32]. A cohort study conducted in Taiwan Province reported that age older than 70 years, male, living in rural areas, diabetes, congestive heart failure, chronic obstructive pulmonary disease, chronic kidney disease and cancer were independent risk factors for incident TB among elderlies. This also highlighted the impact of TB co-morbidity among elderlies [33–35]. However, we did not identify diabetes as a risk factor for developing TB disease among our study population, as reported by studies conducted in Mexico [36] and Korea [37], and also in Taiwan Province of China [27]. There were several reasons underlay it. Firstly, the percentage of diabetes reported among our elderly cohort was 7.0%, which was much lower than reported prevalence, that is, diabetes prevalence being 20.2% in Chinese people aged 60 years and above, and only 36.5% of diabetes patients being aware of their diagnosis [38], resulting in under-reporting of diabetes. Secondly, in our study, diabetes status was mainly self-reported by participants at baseline, we did not perform clinical measurement. Moreover, about half of known diabetes patients lost to follow up during follow-up period.

To our knowledge, this is the first study that investigated the contribution of risk factors to incident TB among elderlies in China. We obtained PAF of each risk factor identified in our study to incident TB among elderly. A study conducted in 22 high TB burden countries mostly in Africa and Asia reported that the top three contribution risk factors were malnutrition (27.0%), smoking (21.0%) and HIV infection (16.0%) [39]. In our study, the biggest contributor for elderly developing bacteriologically positive TB was ex−/current smoker followed by low BMI. For developing active TB, the biggest contributors were non-Han nationality, male sex and smoking. This finding provide the basic data for evaluating the impact of different intervention strategy, and has an important public health implication. It will be very useful for policy maker, especially in resource limited regions.

We observed a high proportion of lost-to-follow-up over the two-year study period, inevitably producing effect on results. In our study, the main reason of high lost-to-follow-up was that participants were healthy elderlies without TB, resulting in a high proportion (about 30%) of them refused the follow-up check. High proportion of lost-to-follow-up was also reported by other cohort studies, even if participants were TB patients. A community-based cohort study conducted among people aged older than 14 years in Southern Ethiopia reported about 1.0% lost-to-follow-up during the first half year over the follow-up period [34], and 6.9% of TB patients lost to follow-up during 2 years was reported by a study conducted in Colombian prisons [40]. In older TB patients, the proportion of death and lost-to-follow-up hit 12.3% and transfer-out was 3.9%, indicated by a study conducted in India [41]. Another reason was that about 8.1% of participants was internal migrants who moved around after living in a place for some time, and 77% of them lost to follow up. In addition, another 5% of our study population was transferred out during the follow-up period.

As a well-designed study with a large number of participants in China, our study has the following strengths. Firstly, it was designed as a prospective cohort study that captured incident TB cases among our cohort and obtained the true TB incidence by performing regular CXR in the follow-up period. Secondly, our study was a random sampled population-based study. It avoided the selection bias existing such as in hospital-based studies, which possibly resulted in a high yield of incident cases as people with risk factors for TB are likely visiting hospitals. For example, in two pilot studies conducted in China to screen TB among diabetes patients, the clinic based study yielded higher TB notification than the community one [42, 43]. Finally, our study had a very strict quality control including internal quality control measurements and external supervision for study procedure and data collection to ensure the study quality.

Our study has several limitations. First, a high proportion of lost-to-follow-up presented among the elderlies with TB risk factors. This might result in an underestimation of TB incidence among elderlies. Second, due to the restriction of funding and resources, we could only conduct a two-year follow-up. However, TB is a chronic infectious disease and the onset of disease took time and only happened once immunity weakened. Two-year follow up was therefore not long enough to capture all TB incidences. We recommend longer follow up among the elderlies in future studies.

Although with some limitations, in the context of ending TB, our population-based prospective cohort study reported a very important area in TB control in a country with high TB burden. By investigating the TB incidence among elderly in China, we provided scientific evidence for the urgent need in conducting active case finding among elderly, a group of TB patients who often do not present TB symptoms. The PAF for TB identified the key population to implement intervention strategies in order to rapidly decrease the TB incidence rate. Based on our study, we could further observe the long term impact of active case finding on local TB epidemic among elderlies and further provide the optimal screening strategy, to reach the first milestone and the targets set in the End TB Strategy.

## Conclusions

From our finding, we conclude that the elderly population in China had a high TB incidence rate and risk to develop TB disease. Given the nature of elderly TB, active case finding should be applied among elderlies to detect more TB cases. Our findings from China, a country with high TB burden also could be referred by other countries with aging population and high TB burdens in order to achieve the targets set in the End TB Strategy.

## Data Availability

The National Center for Tuberculosis Control and Prevention (NCTB) is the custodian of the data for this study. The data are not accessible online, but may be made available upon written request to the NCTB through the authors, if in line with the Ethical Review Board guidelines.

## Abbreviations

BMI: Body mass index
*CI*: Confidence interval
CXR: Chest X-ray
HR: Hazard ratio
NTP: National TB Program
PAF: Population attributable fraction
PY: Person-year
TB: Tuberculosis
WHO: World Health Organization

## References

[1] World Health Organization. World report on aging and health. Available from: https://apps.who.int/iris/bitstream/handle/10665/186463/9789240694811_eng.pdf?sequence=1. Accessed 8 Aug 2019.

[2] World Health Organization. WHO report 2007 Global tuberculosis control: surveillance, planning, financing. Available from: https://apps.who.int/iris/bitstream/handle/10665/43629/9789241563141_eng.pdf?sequence=1. Accessed 8 Aug 2019.

[3] World Health Organization (2017). Global tuberculosis report.

[4] Davies PD (2007). TB in the elderly in industrialised countries. Int J Tuberc Lung Dis.

[5] Wang L, Zhang H, Ruan Y, Chin DP, Xia Y, Cheng S (2014). Tuberculosis prevalence in China, 1990-2010: a longitudinal analysis of national survey data. Lancet.

[6] World Health Organization. China country assessment report on ageing and health. Available from: https://apps.who.int/iris/bitstream/handle/10665/194271/9789241509312_eng.pdf?sequence=1. Accessed 8 Aug 2019.

[7] Pratt RH, Winston CA, Kammerer JS, Kammerer JS, Armstrong LR (2011). Tuberculosis in older adults in the United States, 1993-2008. J Am Geriatr Soc.

[8] Technical Guidance Group of the Fifth National TB Epidemiological Survey, the Office of the Fifth National TB Epidemiological Survey (2012). The fifth national tuberculosis epidemiological survey in 2010. Chin J Antituberc.

[9] Huynh GH, Klein DJ, Chin DP, Wagner BG, Eckhoff PA, Liu R (2015). Tuberculosis control strategies to reach the 2035 global targets in China: the role of changing demographics and reactivation disease. BMC Med.

[10] Mori T, Leung CC (2010). Tuberculosis in the global aging population. Infect Dis Clin N Am.

[11] Vynnycky E, Borgdorff MW, Leung CC, Fine PEM (2008). Limited impact of tuberculosis control in Hong Kong: attributable to high risks of reactivation disease. Epidemiol Infect.

[12] Wang L, Liu X, Huang F, Hennig C, Uplekar M, Jiang S (2010). Engaging hospitals to meet tuberculosis control targets in China: using the internet as a tool to put policy into practice. Bull World Health Organ.

[13] Huang F, Cheng S, Du X, Chen W, Scano F, Falzon D (2014). Electronic recording and reporting system for tuberculosis in China: experience and opportunities. J Am Med Inform Assoc.

[14] Li T, Shewade HD, Soe KT, Rainey JJ, Zhang H, Du X (2019). Under-reporting of diagnosed tuberculosis to the national surveillance system in China: an inventory study in nine counties in 2015. BMJ Open.

[15] Tao L, Chen P, Chen W, Xia Y, Chen H, Zhang H (2016). Assess the sensitivity of China’s TB surveillance system through inventory study and capture-remark-recapture method. Chin J Antitubec.

[16] Cheng J, Wang L, Zhang H, Xia Y (2015). Diagnostic value of symptom screening for pulmonary tuberculosis in China. PLoS One.

[17] Zhang C, Zhao F, Xia Y, Yu Y, Shen X, Lu W, et al. Prevalence and risk factors of active pulmonary tuberculosis among elderly people in China: a population based cross-sectional study. Infect Dis Poverty. 2019;8(7). 10.1186/s40249-019-0515-y. 10.1186/s40249-019-0515-y PMC6337869 30654836

[18] Chen W, Shu W, Wang M, Hou Y, Xia Y, Xu W (2013). Pulmonary tuberculosis incidence and risk factors in rural areas of china: a cohort study. PLoS ONE.

[19] Xiao D, Zhao M, Wang Y, Wang L, Xu S, Wang W (2009). Guidelines for implementing the national tuberculosis control program in China (2008).

[20] National Health Commission of the People’s Republic of China. Criteria of weight for adults: National Health Commission of the People’s Republic of China. Available from: http://www.nhc.gov.cn/ewebeditor/uploadfile/2013/08/20130808135715967.pdf. Accessed 8 Aug 2019.

[21] World Health Organization. The End TB Strategy. Available from: https://www.who.int/tb/End_TB_brochure.pdf?ua=1. Accessed 8 Aug 2019.

[22] Zhu S, Xia L, Yu S, Chen S, Zhang J (2017). The burden and challenges of tuberculosis in China: findings from the global burden of disease study 2015. Sci Rep.

[23] Avilov KK, Romanyukha AA, Borisov SE, Belilovsky EM, Nechaeva OB, Karkach AS (2015). An approach to estimating tuberculosis incidence and case detection rate from routine notification data. Int J Tuberc Lung Dis.

[24] Hochberg NS, Horsburgh CR (2013). Prevention of tuberculosis in older adults in the United States: obstacles and opportunities. Clin Infect Dis.

[25] Hauer B, Brodhun B, Altmann D, Fiebig L, Loddenkemper R, Haas W (2011). Tuberculosis in the elderly in Germany. Eur Respir J.

[26] Nanoo A, Izu A, Ismail NA, Ihekweazu C, Abubakar I, Mametja D (2015). Nationwide and regional incidence of microbiologically confirmed pulmonary tuberculosis in South Africa, 2004-12: a time series analysis. Lancet Infect Dis.

[27] Yen YF, Pan SW, Su VYF, Chuang PH, FengJY SWJ (2018). Influenza vaccination and incident tuberculosis among elderly persons, Taiwan. Emerg Infect Dis.

[28] Negin J, Abimbola S, Marais BJ (2015). Tuberculosis among older adults - time to take notice. Int J Infect Dis.

[29] Marengoni A, Angleman S, Melis R, Mangialasche F, Karp A, Garmen A (2011). Aging with multimorbidity: a systematic review of the literature. Ageing Res Rev.

[30] Leung CC, Yew WW, Chan CK, Chau CH, Tam CM, Lam CW (2002). Tuberculosis in older people: a retrospective and comparative study from Hong Kong. J Am Geriatr Soc.

[31] Leung CC, Li T, Lam TH, Yew WW, Law WS, Tam CM (2004). Smoking and tuberculosis among the elderly in Hong Kong. Am J Respir Crit Care Med.

[32] Jeon CY, Murray MB (2008). Diabetes mellitus increases the risk of active tuberculosis: a systematic review of 13 observational studies. PLoS Med.

[33] Siroka A, Ponce NA, Lonnroth K (2016). Association between spending on social protection and tuberculosis burden: a global analysis. Lancet Infect Dis.

[34] Woldesemayat EM, Datiko DG, Lindtjørn B (2015). Follow-up of chronic coughers improves tuberculosis case finding: results from a community-based cohort study in southern Ethiopia. PLoS One.

[35] Lönnroth K, Williams BG, Cegielski P, Dye C (2010). A consistent log-linear relationship between tuberculosis incidence and body mass index. Int J Epidemiol.

[36] Ponce-De-Leon A, Garcia-Garcia MDL, Garcia-Sancho MC, Gomez Perez FJ, Valdespino Gomez JL, Olaiz Fernandez G (2004). Tuberculosis and diabetes in southern Mexico. Diabetes Care.

[37] Kim SJ, Hong YP, Lew WJ, Yang SC, Lee EG (1995). Incidence of pulmonary tuberculosis among diabetics. Tuber Lung Dis.

[38] Wang L, Gao P, Zhang M, Huang Z, Zhang D, Deng Q (2017). Prevalence and ethnic pattern of diabetes and prediabetes in China in 2013. JAMA.

[39] Lönnroth K, Castro KG, Chakaya JM, Chauhan LS, Floyd K, Glaziou P (2010). Tuberculosis control and elimination 2010-50: cure, care, and social development. Lancet.

[40] Rueda ZV, López L, Vélez AL, Marín D, Giraldo MR, Pulido H (2013). High incidence of tuberculosis, low sensitivity of current diagnostic scheme and prolonged culture positivity in fore Colombian prisons, a cohort study. PLoS One.

[41] Oshi D, Oshi SN, Alobu I, Ukwaja KN (2014). Profile and treatment outcomes of tuberculosis in the elderly in southeastern Nigeria, 2011–2012(R20). PLoS One.

[42] Lin Y, Innes A, Xu L, Li L, Chen J, Hou J (2015). Screening of patients with diabetes mellitus for tuberculosis in community health settings in China. Trop Med Int Heal.

[43] Lin Y, Li L, Mi F, Du J, Dong Y, Li Z (2012). Screening patients with diabetes mellitus for tuberculosis in China. Tropical Med Int Health.

